# Delayed capsular bag distension syndrome

**DOI:** 10.4103/0974-620X.71905

**Published:** 2010

**Authors:** Kalyan Das

**Affiliations:** Department of Cornea, Cataract and Refractive Surgery, Sri Sankaradeva Nethralaya, Guwahati, Assam - 781028, India

Capsular bag distension syndrome (CBDS) is a complication of continuous curvilinear capsulorhexis (CCC)[1–3] done in phacoemulsification and in the bag IOL implantation.

CBDS occurs mostly due to accumulation of turbid fluid behind the IOL with or without refractive change. It has been classified according to the time of onset, intra-operative, early post-operative and late post-operative.[4]

In late post operative CBDS, the margin of the CCC is blocked by the IOL optic and this produces a closed chamber inside the capsular bag in which the turbid fluid accumulates. This phenomenon is also known as liquefied after cataract[5,6] or capsulorhexis - related lacteocrumenasia.[7]

After Nd:YAG laser capsulotomy, the fluid usually disappears immediately and patient’s symptomatology improves.

**Figure 1:**
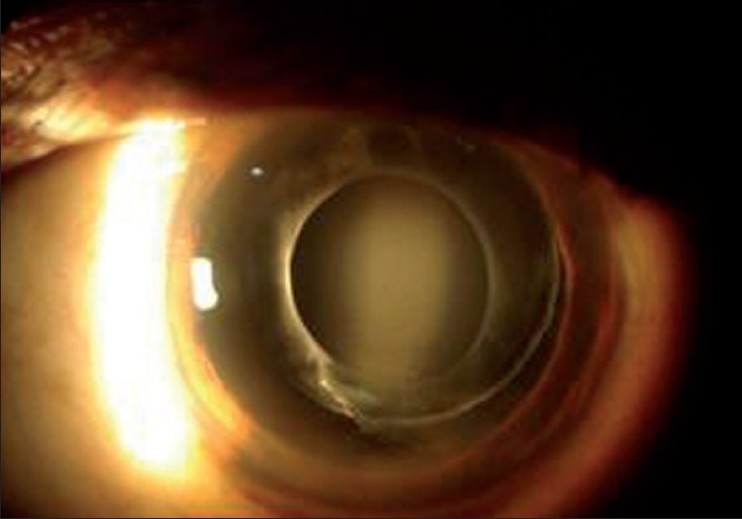
Pre treatment - Accumulation of turbid fluid in the space between IOL and posterior capsule

**Figure 2:**
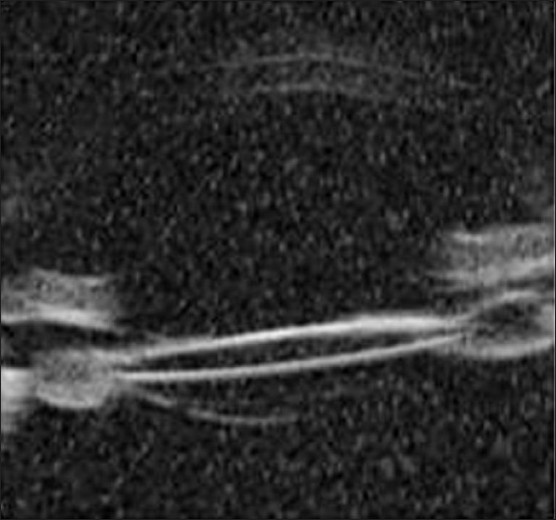
Pre treatment UBM - UBM showing in the bag IOL and posterior bowing of posterior capsule

A 55-years-old male patient underwent uneventful clear corneal phacoemulsification and in-the-bag IOL implantation (Aurolab, Madurai, India) in the right eye (OD). Medical records showed postoperative visual acuity of 6/6, N6 OD. He presented to us three years after surgery with complaints of mild dimness of vision (6/12, N8) and seeing cob-web like matter with his right eye of two weeks duration. Slit lamp examination OD showed a normal anterior chamber, and a well centered in-the-bag IOL. There was posterior bowing of the posterior lens capsule with accumulation of turbid fluid in the space between the IOL and the capsule [Figure 1]. Ultrasound biomicroscopic examination (UBM) confirmed the findings [Figure 2]. The intraocular pressure was 18 mm of Hg in both eyes. Fundus examination was normal.

**Figure 3:**
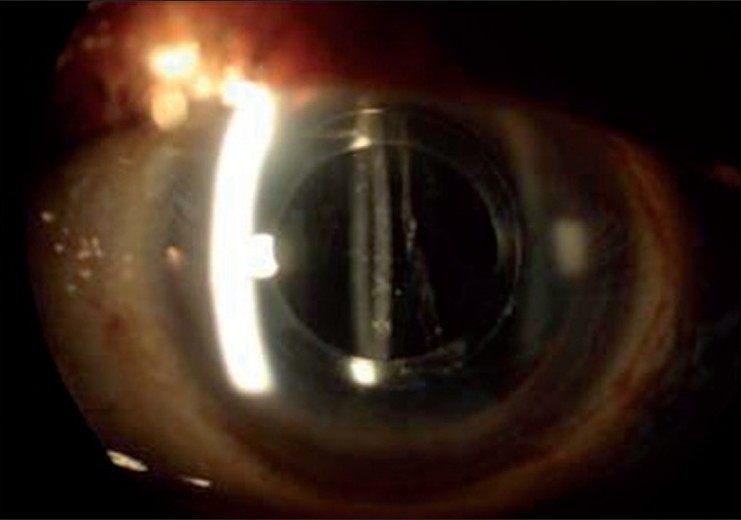
Post treatment - Following YAG laser capsulotomy disappearance of turbid fluid

**Figure 4:**
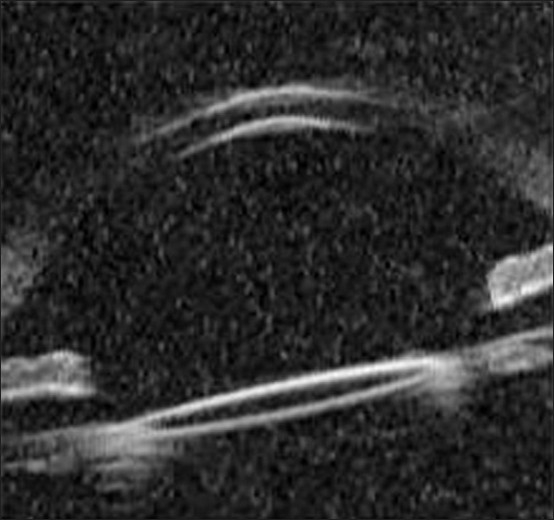
Post treatment UBM - UBM confirming disappearance of retro IOL space following YAG laser

**Figure 5:**
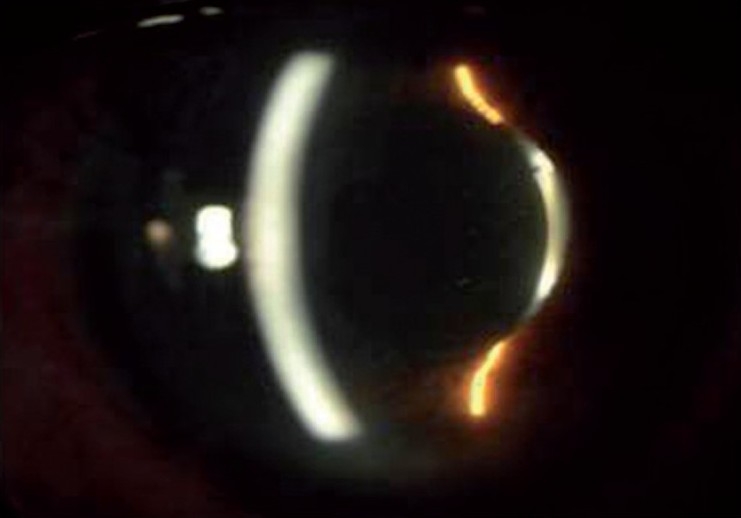
Turbid fluid is seen percolating into anterior chamber following YAG laser treatment

A diagnosis of CBDS was made and Nd:YAG laser capsulotomy performed on the anterior lens capsule beyond the edge of the IOL optic in the most dependant position. The CBDS resolved immediately with leakage of the turbid fluid into the anterior chamber [Figure 3–5]. Post operatively, he was treated with two hourly Betnesol-N eye drops (GlaxoSmithKline) for two weeks. This was tapered over two weeks. Patient clinically and symptomatically improved. Visual acuity after two weeks was 6/6, N.6 and there was complete disappearance of the visual disturbances.

CBDS is a rare complication of cataract surgery and in the bag IOL implantation with CCC.[8] Decrease in visual acuity is due to accumulation of turbid fluid inside the distended capsular bag or shift in the refractive error toward myopia[1–3] and or hyperopia.[9]

Residual epithelial cells undergo metaplasia and proliferate, producing numerous types of collagen and extracellular matrix that accumulate in the capsular bag.[8] Nd:YAG laser capsulotomy of the anterior capsule leads to leakage of the fluid into anterior chamber which is then drained through aqueous drainage pathway.

Awareness about this syndrome is important for a cataract surgeon which helps in proper management of this condition.
